# The UF-5000 Atyp.C parameter is an independent risk factor for bladder cancer

**DOI:** 10.1038/s41598-024-63572-0

**Published:** 2024-06-03

**Authors:** Tong Zhang, Jianhong Zhu, Zhaoxing Li, Ya Zhao, Yan Li, Jing Li, Qian He, Yan Geng, Wei Lu, Lei Zhang, Zhenzhen Li

**Affiliations:** Department of Clinical Laboratory, Second Hospital, Xi’an Jiaotong University, Xi’an, 710004 China

**Keywords:** Bladder cancer, Atyp.C, Conditional logistic regression, Risk factor, Health occupations, Oncology, Risk factors, Urology

## Abstract

Bladder carcinoma (BC) accounts for > 90% of all urothelial cancers. Pathological diagnosis through cytoscopic biopsy is the gold standard, whereas non-invasive diagnostic tools remain lacking. The “Atyp.C” parameter of the Sysmex UF-5000 urine particle analyzer represents the ratio of nucleus to cytoplasm and can be employed to detect urinary atypical cells. The present study examined the association between urinary Atyp.C values and BC risk. This two-center, retrospective case–control study identified clinical primary or newly recurrent BC (study period, 2022–2023; n = 473) cases together with controls with urinary tract infection randomly matched by age and sex (1:1). Urinary sediment differences were compared using non-parametric tests. The correlations between urinary Atyp.C levels and BC grade or infiltration were analyzed using Spearman’s rank correlation. The BC risk factor odds ratio of Atyp.C was calculated using conditional logistic regression, and potential confounder effects were adjusted using stepwise logistic regression (LR). Primary risk factors were identified by stratified analysis according to pathological histological diagnosis. The mean value of urinary Atyp.C in BC cases (1.30 ± 3.12) was 8.7 times higher than that in the controls (0.15 ± 0.68; *P* < 0.001). Urinary Atyp.C values were positively correlated with BC pathological grade and invasion (r = 0.360, *P* < 0.001; r = 0.367, *P* < 0.001). Urinary Atyp.C was an independent risk factor for BC and closely related with BC pathological grade and invasion. Elevated urinary Atyp.C values was an independent risk factor for BC. Our findings support the use of Atyp.C as a marker that will potentially aid in the early diagnosis and long-term surveillance of new and recurrent BC cases.

## Introduction

Bladder carcinoma (BC) is the most common urothelial malignancy, with high incidence and recurrence rates^1,2^. The gold standard for BC diagnosis is biopsy via cystoscopy, a painful, costly, and invasive type of endoscopy that places a heavy burden on both patients and healthcare providers^3,4^. Risk factors for BC development include cigarette smoking and exposure to cyclic chemicals, dyes, rubbers, textiles, and paints. Non-invasive evaluation using computed tomography (CT) revealed that bladder occupation had a suggestive effect on the early BC diagnosis, whereas the early suggestive effect of the clinical test method is unreported^4,5^.

Because the bladder is the exclusive reservoir organ for urinary cells, urinary sediments provide a source of exfoliated bladder tumor cells^6^. Using urinary samples to explore BC-related factors is cost-effective and non-invasive. Traditional urine cytology remains a mandatory screening method for patients at high risk of urothelial carcinoma in clinical practice^7,8^. The diagnostic accuracy depends on the sample type and adequacy, treatment method, underlying clinical conditions, tumor grade, and level of morphological knowledge of the examiner. In reality, the diagnostic accuracy can additionally be impacted by the presence of poorly preserved cells, inflammation, infection, and a small number of cells with irregular morphology. Thus, it can be difficult for cytopathologists to make a definitive diagnosis. The ambiguous diagnostic category of “atypical cells” has caused great distress to cytopathologists and clinicians^9,10^. Moreover, urine cytology is a time-consuming and wearisome examination^11^. Given such shortcomings, its efficacy in BC screening is greatly reduced^11^. Hence, exploring more simple and convenient urine screening markers for BC that are accurate and do not increase patient burden is necessary.

The Sysmex UF-5000 uses fluorescence flow cytometry and hydrodynamic focusing for urine sediment analysis. Intracellular particles and nucleic acids are stained with specific fluorescent dyes and then stimulated by a semiconductor laser to produce different scattered light and fluorescence signals^12,13^. The counting and classification of urine cells are based on such signals to determine particle characteristics^14^. The Sysmex UF-5000 provides a new parameter to distinguish atypical cells, Atyp.C, which results from differences in the fluorescent staining of nucleic acids in urothelial cells using a lateral fluorescent signal waveform region. Atypical cells exhibit lateral fluorescence and scattered light characteristics, indicating nuclear enlargement and increased nucleo-plasmic ratios^14^. The “Atyp.C” parameter indicates the number of atypical/malignant urothelial cells with abnormally high levels of nucleic acid content, providing an important indicator for BC diagnosis, monitoring, and treatment.

Recent studies of human urothelial cancers have demonstrated that “Atyp.C” can identify cancer cells with a 71.1% agreement with urine cytology, which has some auxiliary significance in the clinical diagnosis of urothelial carcinoma^15,16^. The purpose of this study was to explore the relationship between Atyp.C and BC risk in detail, which should lay a foundation for the later optimization of Atyp.C as an early non-invasive diagnostic and monitoring indicator of BC.

## Methods

Urinary Atyp.C levels were compared between patients clinically diagnosed with BC (cases) and individuals without BC (controls) in this case–control study. This study was performed in line with the principles of the Declaration of Helsinki. The Research Ethics Committee of the University of Medical Sciences, the Second Affiliated Hospital of Medical School of Xi’an Jiaotong University (Approval No.: 2023469), approved this retrospective study and waived the need for obtaining informed consent. Conditional logistic regression analysis was employed to derive the odds ratios (OR) for BC. The relationship between urinary Atyp.C and BC type was analyzed according to pathological classification.

### Case–control population selection

Patients with a first clinical diagnosis or new recurrence of BC between March 1, 2022, and May 30, 2023, were included in the case groups, whereas cases with benign and preoperative chemotherapy were excluded. The control groups, composed of patients with urinary tract infection, were age‐ and sex‐matched to cases at a 1:1 ratio by random sampling, applying the same exclusion criteria as for the cases: tumor, tumor tendency, and tumor disease history. Clinical information about the cases was extracted from the electronic medical record of the two large 3A hospitals in Xi’an. These platforms ensured the authenticity and validity of the case material.

### Statistical analysis

Statistical analyses were performed using IBM SPSS Statistics version 21.0 and the stats package, and plots were constructed with the ggplot2 package in R (version 4.2.1). Two-sided *P*-values < 0.05 were considered as statistically significant. The Mann–Whitney U test was used to analyze urinary Atyp.C and non-squamous epithelial cell (N.SEC), squamous epithelial cell (SEC), and white blood cell (WBC) count differences between the cases and controls. The significance of the difference in the distribution of Atyp.C and N.SEC, SEC, and WBC counts among pathological types was evaluated with the Kruskal–Wallis test. The correlation between urinary Atyp.C values and pathological grades and invasion was assessed using Spearman’s rank correlation. The OR for urinary Atyp.C as a risk factor for BC was calculated using conditional logistic regression analysis, and the effects of potential confounders such as SEC, N.SEC, WBC, occult blood (BLD), leukocyte esterase (LEU), protein (PRO), and urinary color (COL) and turbidity (CLA) were adjusted by stepwise logistic regression.

### Ethical approval

This study has been approved by the Research Ethics Committee of the University of Medical Sciences, the Second Affiliated Hospital of Medical School of Xi’an Jiaotong University (Approval No.: 2023469).

## Results

### Summary of the baseline characteristics

**Figure 1:**
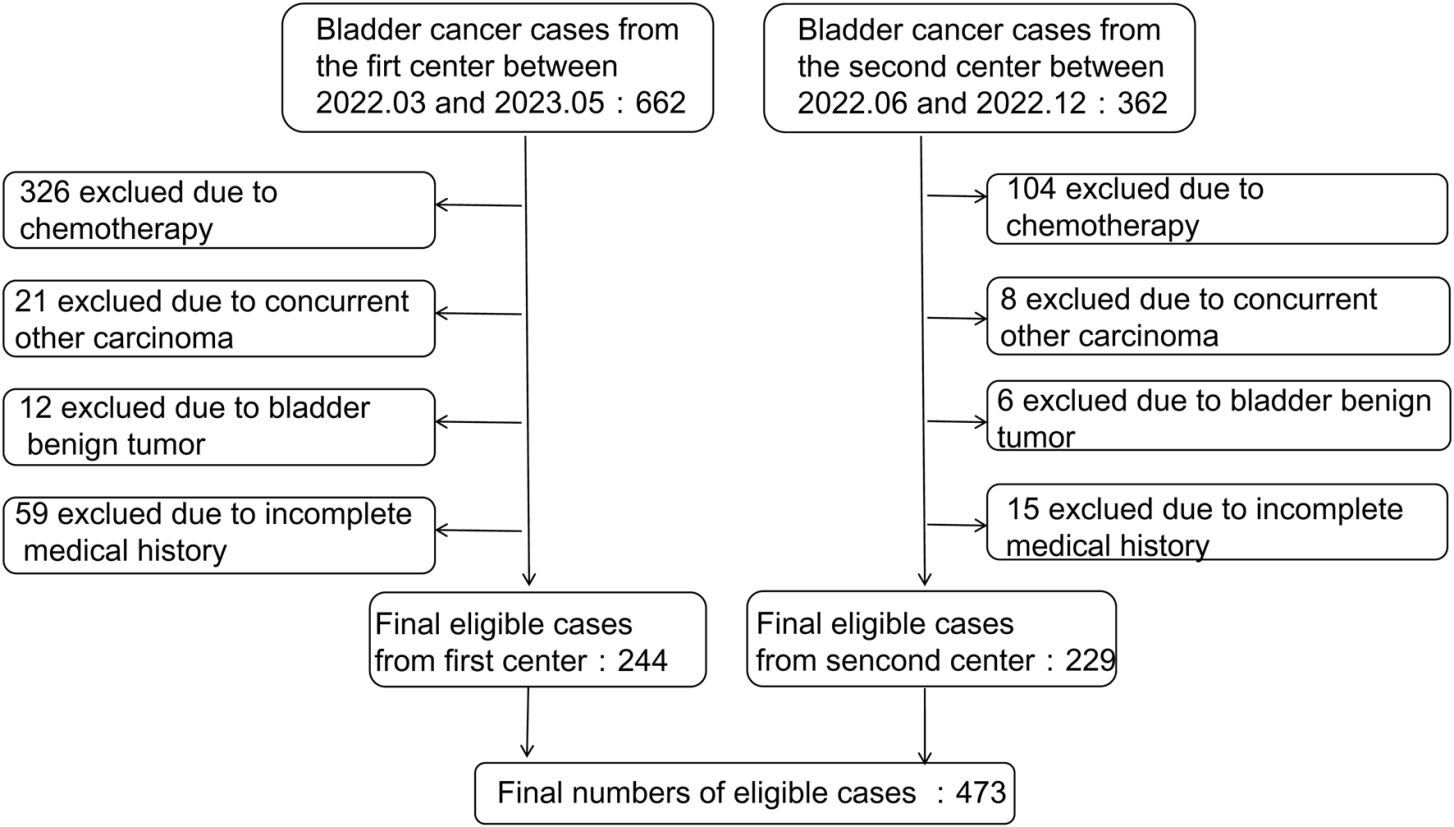
Study profile.

A total of 1024 BC cases were examined between March 1, 2022, and May 30, 2023. After all inclusion and exclusion criteria were applied, 473 cases remained (Fig. 1), including 314 (66.4%) new and 154 (33.6%) recurrent cases; histopathological results were well-established in 237 cases. This dataset consisted of 20.2% high-grade muscle-invasive BC (HMIBC) cases, 14.3% high-grade non-muscle-invasive BC (HNMIBC) cases, 3.7% low-grade muscle-invasive BC (LMIBC) cases, 26.5% low-grade non-muscle-invasive BC (LNMIBC) cases, 12.2% papillary urothelial neoplasm of low malignant potential (PUNLMP) cases, 11.8% papillary urothelial carcinoma grade II BC (PUCII) cases, and 10.9% cases of other pathologic types of BC (including carcinosarcoma, superficial bladder tumors, and spindle-shaped tumors). The population comprised 75.4% males and 24.6% females with a median age of 67 [interquartile range (IQR): 59, 75] years. Although no significant differences were found in age and sex (*P* > 0.05), urinary sediment analysis results showed different distributions between cases and controls (Table 1).

**Table 1:** Baseline characteristics of cases and controls.

Data	Cases (n = 473)	Controls (n = 473)	*P* value
Gender n (%)			
Male	357 (75.4%)	357 (75.4%)	> 0.5
Female	116 (24.6%)	116 (24.6%)	> 0.5
Age, years			
Median (IQR)	67 (59, 75)	67 (59, 75)	> 0.5
Blandder cancer n (%)			
The first clinical diagnosis	314 (66.4%)		
The new recurrence	154 (33.6%)		
Results of urine sediment			
Atyp.C (Median (IQR)) /ul	0.2 (0, 0.9)	0 (0, 0.1)	0.000
N.SEC (Median (IQR))/ul	4.1 (1.4, 10.6)	2.3 (0.9, 5.8)	0.000
SEC (Median (IQR))/ul	6.9 (2.4, 17.8)	4.4 (1.6, 10.0)	0.000
WBC (Median (IQR))/ul	88.2 (19.4, 311.7)	50.6 (8.3, 301.8)	0.02
Results of dipstick urinalysis			
BLD n (%)			
BLD (−)	70 (14.8%)	138 (29.2%)	
BLD (±)	39 (8.2%)	62 (13.1%)	
BLD (1+)	57 (12.1%)	72 (15.2%)	
BLD (2+)	45 (9.5%)	65 (13.7%)	
BLD (3+)	262 (55.4%)	136 (28.8%)	
LEU n (%)			
LEU (−)	212 (44.8%)	196 (41.4%)	
LEU (±)	41 (8.7%)	28 (5.9%)	
LEU (1+)	75 (15.9%)	80 (16.9%)	
LEU (2+)	62 (13.1%)	57 (12.1%)	
LEU (3+)	83 (17.5%)	112 (23.7%)	
PRO n (%)			
PRO (−)	160 (33.8%)	264 (55.8%)	
PRO (±)	90 (19%)	79 (16.7%)	
PRO (1+)	94 (19.9%)	70 (14.8%)	
PRO (2+)	85 (18%)	40 (8.5%)	
PRO (3+)	44 (9.3%)	20 (4.2%)	
Col n (%)			
Yellow	378 (79.9%)	443 (93.7%)	
Other	95 (20.1%)	30 (6.3%)	
CLA n (%)			
Clear	312 (66.0%)	373 (78.9%)	
Cloudy	161 (20.1%)	100 (21.1%)	

SEC, squamous epithelial cells; N.SEC, non-squamous epithelial cells; WBC, white blood cell count; BLD, occult blood; LEU, leukocyte esterase; PRO, protein; COL, urinary color; CLA, urinary turbidity; IQR, interquartile range.

### Urinary Atyp.C in BCs

**Figure 2:**
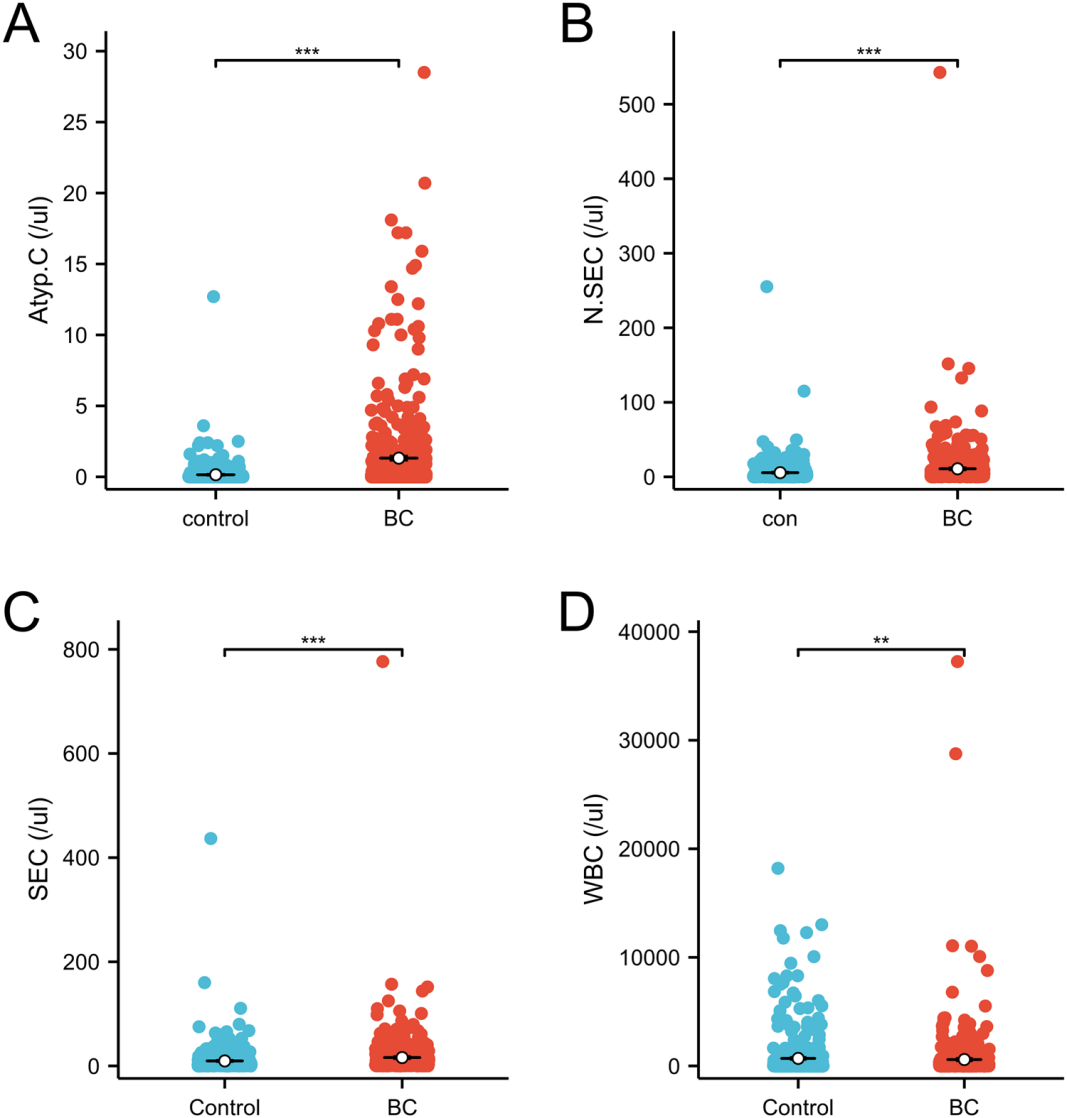
Differences among the distributions of Atyp.C, N.SEC, SEC and WBC in urinary sediment analysis results of cases and controls. N.SEC, non-squamous epithelial cell; SEC, squamous epithelial cell; WBC, white blood cell.

All 473 patients were newly diagnosed or possessed newly relapsed BCs and had no history of other malignancies (Table 1). The median urinary Atyp.C values were 0.2 (IQR: 0, 0.9)/ul and 0 (IQR: 0, 0.1)/ul (*P* < 0.001) in cases and controls, respectively; the median N.SEC counts were 4.1 (IQR: 1.4, 10.6)/ul and 2.3 (IQR: 0.9, 5.8)/ul (*P* < 0.001), respectively, the median SEC counts were 6.9 (IQR: 2.4, 17.8)/ul and 4.4 (IQR: 1.6, 10.0)/ul (*P* < 0.001), respectively, and the median WBC counts were 88.2 (IQR: 19.4, 311.7)/ul and 50.6 (IQR: 8.3, 301.8)/ul (*P* = 0.02), respectively (Fig. 2). Patients with BC presented higher urinary Atyp.C levels than controls.

### Urinary Atyp.C positively correlated with BC histopathological diagnosis

#### Distribution and difference analysis of urinary sediments in different pathologic types of BC

**Figure 3:**
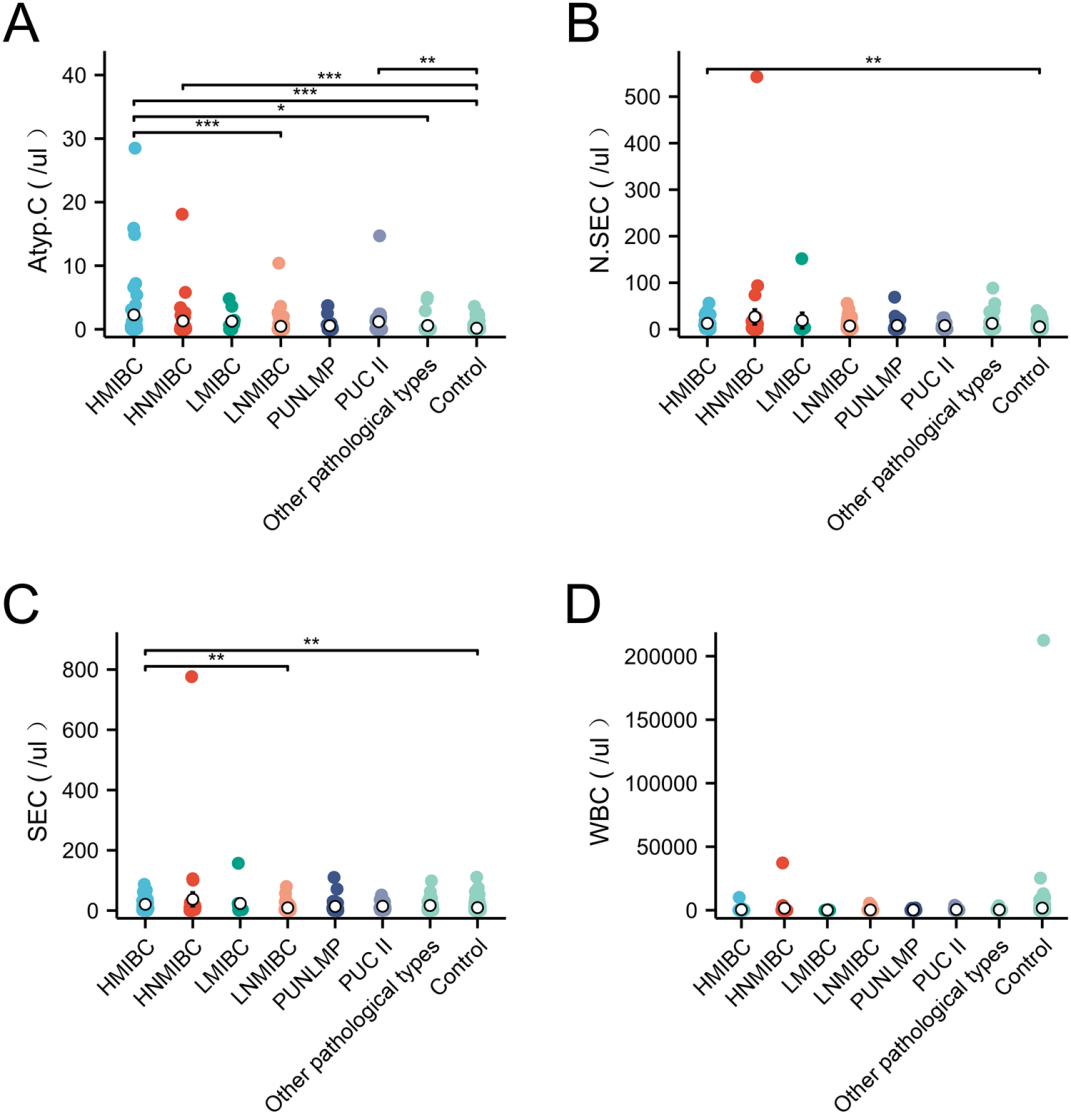
Differences in urinary Atyp.C, N.SEC, SEC and WBC distributions in different pathologic types of BC (***P < 0.001, * *P* < 0.05). N.SEC, non-squamous epithelial cell; SEC, squamous epithelial cell; WBC, white blood cell; BC, bladder carcinoma.

The distribution of urinary Atyp.C, N.SEC, SEC, and WBC were analyzed to account for the correlations between urinary Atyp.C and different pathological BC types. The median urinary Atyp.C gradually reduced, as the pathologic grade went from high to low or invasive to non-invasive,while N.SEC, SEC, and WBC did not show these changes (Table 2). The Kruskal–Wallis test revealed significant differences in urinary Atyp.C, N.SEC and SEC counts across the seven pathological types of BC (*P* < 0.05), except WBC (Fig. 3D). Furthermore, post-hoc testing (Dunnett’s method) revealed that urinary Atyp.C in HMIBC was the highest and was statistically different compared with LNMIBC, other pathological types, and controls (*P* < 0.05). Urinary Atyp.C in the HNMIBC and PUCII group was respectively higher than that controls (*P* < 0.05). Urinary Atyp.C did not differ between HNMIBC, LMIBC, LNMIBC, PUNLMP, PUCII, and other pathological types (*P* > 0.05) (Fig. 3A). There was almost no difference in N.SEC and SEC among the different pathological types of BC, and only a slight difference was observed compared to the controls (*P* < 0.05) (Fig. 3B,C).Thus, the changes in Atyp. C levels were associated with the high grade and infiltration of BC, but N.SEC, SEC, and WBC did not show a corresponding relationship with BC.

**Table 2:** The distribution of urinary Atyp.C, N.SEC, SEC, and WBC in different pathological types of BC (/ul).

Blandder cancer grade	N (%)	Age, years (IQR)	Gender (M/F)	Atyp.C (IQR)	N.SEC (IQR)	SEC (IQR)	WBC (IQR)
HMIBC	48	64 (59, 73)	38/10	0.5 (0.1, 1.53)	8 (2.05, 19.33)	11.35 (5.43, 32.6)	119.5 (34.4, 317.5)
HNMIBC	34	65 (58, 73)	23/11	0.2 (0.1, 1.35)	5.2 (2.23, 10.78)	7.5 (3.38, 16.6)	126.15 (21.33, 546.8)
LMIBC	9	65 (57, 79)	7/2	0.6 (0, 1.4)	1.4 (1.3, 3.1)	3.8 (1.7, 11.9)	26 (15.6, 95.8)
LNMIBC	63	64 (55, 73)	54/9	0 (0, 0.35)	2.9 (1.15, 7.5)	4.4 (1.65, 9.85)	50.9 (11.45, 144)
PUNLMP	29	60 (53, 68)	22/7	0.1 (0, 0.7)	2.4 (1.2, 8.6)	5.3 (1.8, 9.4)	16.1 (4.2, 262.7)
PUCII	28	67 (57, 75)	18/10	0.1 (0, 1.45)	5.1 (2.1, 11.2)	8.25 (3.83, 26.18)	107.85 (41.55, 377.68)
Other pathological types	26	63 (57, 71)	20/6	0.05 (0, 0.275)	3 (1.53, 11.43)	6.95 (2.13, 20.05)	47.75 (22.98, 182.6)
Total	237	64 (57, 73)	182/55	0.1(0, 0.8)	4.4 (1.5, 10.9)	6.7 (2.4, 17.8)	68.6 (18.3, 268.9)

M/F, male/famle; SEC, squamous epithelial cells; N.SEC, non-squamous epithelial cells; WBC, white blood cell; IQR, interquartile range; HMIBC, high-grade muscle invasive bladder cancer; HNMIBC, high-grade non-muscle invasive bladder cancer; LMIBC, low-grade muscle invasive bladder cancer; LNMIBC, low-grade non-muscle-invasive bladder cancer; PUNLMP, papillary urothelial neoplasm of low malignant potential; PUCII, papillary urothelial carcinoma grade II bladder cancer; BC, bladder carcinoma.

#### Correlation between urinary Atyp.C and BC pathological grade and infiltration

**Figure 4:**
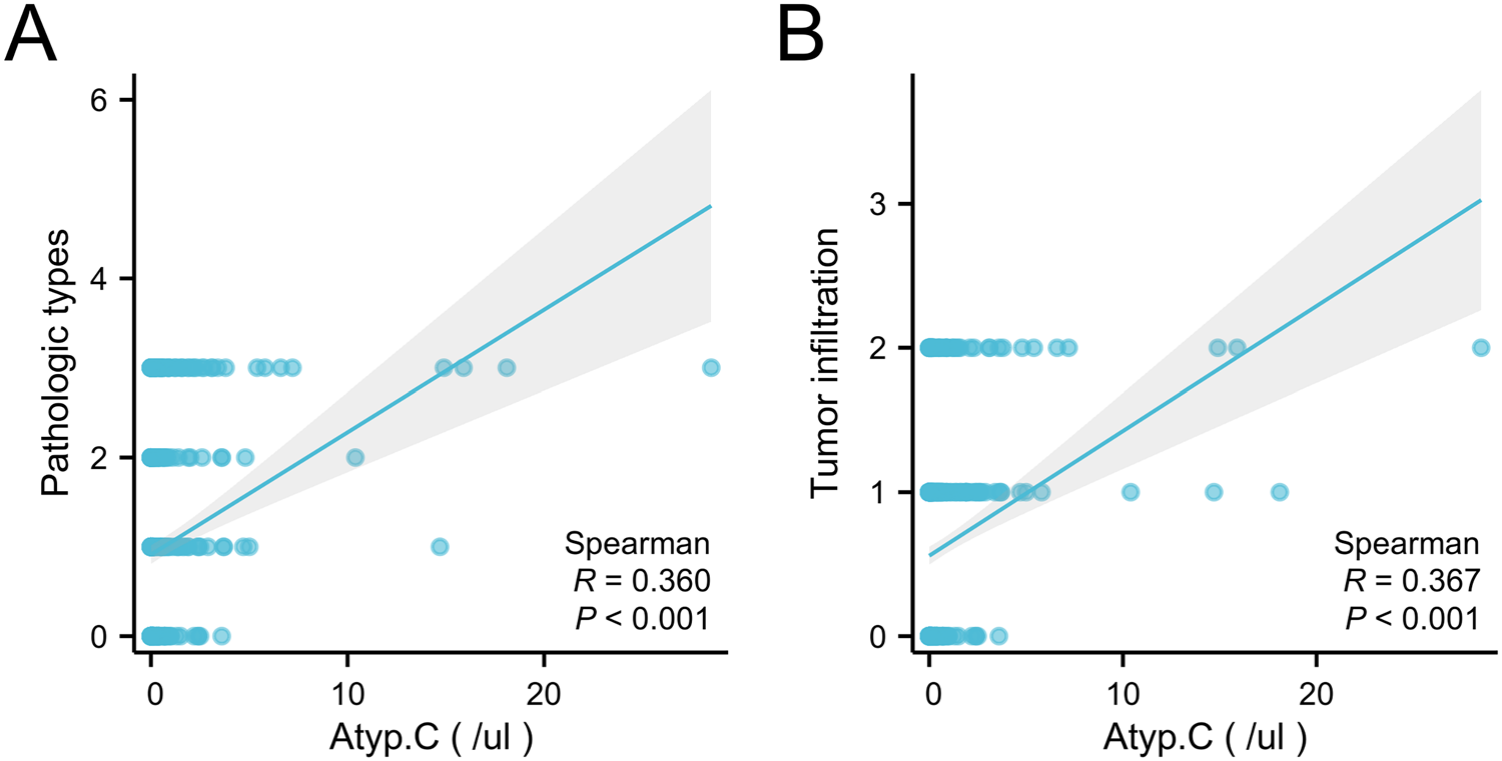
Correlation analysis between urinary Atyp.C and BC pathological grade or infiltration. BC, bladder carcinoma.

The correlation coefficient (r) between urinary Atyp.C levels and pathological grade was 0.360 for controls (*P* < 0.001) and that between urinary Atyp.C values and infiltration was 0.367 for BC (*P* < 0.001). A moderate positive correlation was found between urinary Atyp.C levels and BC pathologic grades and infiltration (Fig. 4).

### Urinary Atyp.C is an independent risk factor for BC

#### Potential risk factors for BC

Random allocation was performed in a 1:1 ratio, with 473 case–control patients matched for age and sex (Table 1). Only factors with *P*-values of < 0.05 in univariable analysis were subsequently estimated with ORs and 95% confidence intervals (CIs) using conditional logistic regression multivariate analysis. The OR was 3.358 (95% CI, 2.33–4.841) for urinary Atyp.C; N.SEC, SEC, BLD (3+), LEU (3+), PRO, COL, and CLA were all risk factors for BC (Table 3). The Atyp.C OR was 3.488 (95% CI, 2.280–5.337), when urinary N.SEC, SEC, BLD, PRO, LEU, COL, and CLA were included in the covariates for step-by-step regression adjustment, and it was stable compared with the baseline model. The ORs for BLD (3+), LEU (3+), and COL were statistically significant and significantly changed compared with the baseline model. The ORs for urinary N.SEC, SEC, PRO, and CLA levels were not statistically significant and were excluded from the new model (Table 3). Urinary Atyp. C, BLD (3+), LEU (3+), and COL levels were identified as risk factors for BC.

**Table 3:** Factors in urinary sediment associated with BC.

	Univariable OR (95% CI)	*P* value	Multivariable OR (95% CI)	*P* value
Atyp.C	3.358 (2.33–4.814)	< 0.001	3.488 (2.28–5.337)	< 0.001
N.SEC	1.026 (1.012–1.040)	< 0.001	–	–
SEC	1.014 (1.006–1.023)	0.001	–	–
WBC	1.000 (1.000–1.000)	0.271	–	–
BLD (−)	1.000 (Ref)	–	1.000 (Ref)	–
BLD (±)	1.116 (0.669–1.861)	0.674	1.137 (0.646–2.003)	0.656
BLD (1+)	1.600 (0.971–2.638)	0.065	1.590 (0.901–2.805)	0.109
BLD (2+)	1.370 (0.838–2.240)	0.209	1.320 (0.762–2.287)	0.322
BLD (3+)	3.968 (2.701–5.829)	< 0.001	2.794 (1.754–4.450)	< 0.001
LEU (−)	1.000 (Ref)	–	1.000 (Ref)	–
LEU (±)	1.420 (0.807–2.501)	0.224	1.242 (0.590–2.613)	0.569
LEU (1+)	0.855 (0.577–1.269)	0.437	0.491 (0.292–0.826)	0.007
LEU (2+)	1.002 (0.650–1.544)	0.994	0.507 (0.285–0.902)	0.021
LEU (3+)	0.699 (0.495–0.986)	0.041	0.316 (0.200–0.502)	< 0.001
PRO (−)	1.000 (Ref)	–	1.000 (Ref)	–
PRO (±)	1.857 (1.285–2.684)	0.001	–	–
PRO (1+)	2.357 (1.587–3.500)	< 0.001	–	–
PRO (2+)	3.648 (2.313–5.754)	< 0.001	–	–
PRO (3+)	3.148 (1.802–5.501)	< 0.001	–	–
Other COL (vs yellow)	3.826 (2.418–6.055)	< 0.001	2.418 (1.353–4.320)	0.003
Cloudy CLA (vs Clear)	1.910 (1.422–2.567)	< 0.001	–	–

SEC, squamous epithelial cells; N.SEC, non-squamous epithelial cells; WBC, white blood cell count; BLD, occult blood; LEU, leukocyte esterase; PRO, protein; COL, urinary color; CLA, urinary turbidity; IQR, interquartile range; BC, bladder carcinoma.

#### Stability of potential risk factors in different BC types

**Figure 5:**
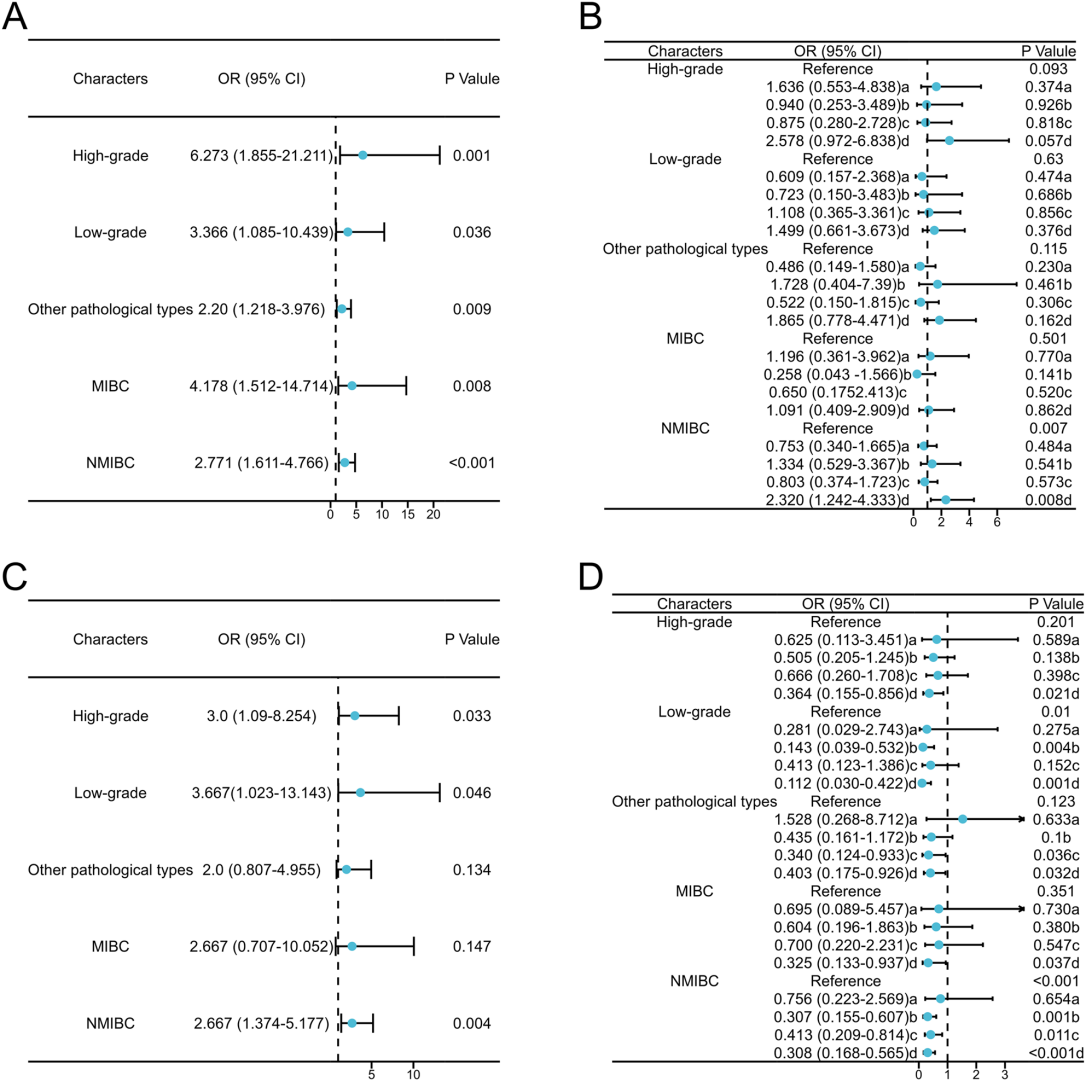
Forest map of ORs of potential risk factors for BC after stratified analysis according to different pathological types. A: Atyp.C; B: BLD (with a:BLD(±); b: BLD(1+); c: BLD(2+); d:BLD(3+)); C: COL; D: LEU (with a: LEU(±); b: LEU(1+); c: LEU(2+); d: LEU(3+)).

The stability of the risk factors in different BC pathological types was further assessed by dividing the 237 cases with complete pathological information into five categories: high-grade BC, low-grade BC, other BCs (including PUNLMP, PUCII, and other pathological types), invasive BC, and non-invasive BC. Stratified analysis of pathological classification using conditional logistic analysis showed that only urinary Atyp.C levels significantly correlated with BC in each type; higher urinary Atyp.C values correlated with higher ORs and a higher degree of pathological grade and invasion (Fig. 5A). Stratified analysis revealed that the correlation between BLD (3+), LEU (3+), and COL was greatly reduced with the occurrence of each BC pathological type (Figs. 5B–D). In summary, elevated urinary Atyp.C levels were an independent risk factor for BC.

## Discussion

The greatest obstacles to BC treatment are the high tumor recurrence rates and lack of non-invasive, inexpensive, and sensitive specific biological markers for tumor monitoring^17,18^. Over the past decades, the limitations of cystoscopy, including invasiveness and high cost, have led to the development of several urinary-based diagnostic methods for BC surveillance, including urinary cytology^10,19^, fluorescence in situ hybridization^20,21^, urinary protein detection (BTA-STAT)^22,23^, NMP22^24–26^, and others^27^. However, all these biomarkers have limitations and must be combined with others for successful malignancy monitoring, which adds to the financial burden on patients^27^. This study expected to screen more risk factors from routine inspection that could detect early BC stages to help quicken diagnosis and treatment, avoid missed diagnoses, and reduce the burden on patients.

Urinary Atyp.C is a parameter used in UF-5000 that is measured using a non-invasive, fast, and inexpensive assay. As atypical/malignant cells tend to exhibit excessive chromatin proliferation, the dye assessed by UF-5000 urinary samples can cross the cell membrane and bind to nucleic acids, leading to differences in the fluorescent staining of uroepithelial cells based on the nucleic acid content. Atypical cells with abnormally high nucleic acid levels can be detected using the lateral fluorescent signal waveform region. This technique could be a key tool for the early diagnosis and monitoring of BC.

Urinary Atyp.C has been employed to diagnose urothelial carcinoma, with a 59.0% sensitivity, 82.1% specificity, 75.0% positive predictive value, 68.8% negative predictive value, and 71.1% coincidence rate with urinary cell exudation^16^. These results show that urinary Atyp.C analysis can indicate the number of typical/malignant urothelial cells, which is consistent with urinary Atyp.C being an independent risk factor for BC. Despite these findings, their sample size was small, and the specific cutoff range was not determined. The current study increased the sample size and found that urinary mean Atyp.C levels in BC (1.30 ± 3.12) were 8.7 times higher than those in controls without BC (0.15 ± 0.68). Levels were additionally higher in high-grade and/or invasive BC, which is consistent with the conclusion of Ren et al. that urinary Atyp.C intensity may be a good indicator of histological diagnosis of high-grade urothelial carcinomas^16^. The present study found that urinary Atyp.C values positively correlated with tumors’ pathologic types (r = 0.360) and infiltration (r = 0.367). Urinary Atyp.C, N.SEC, SEC, BLD (3+), LEU (3+), PRO, COL, and CLA were identified as risk factors for BC with univariate or age- and sex-adjusted ORs estimation using logistic regression analysis. The OR for urinary Atyp.C was 3.358 (95% CI 2.33–4.814). Only urinary Atyp.C, BLD (3+), LEU (3+), and COL were statistically significant after multiple stepwise regression analysis on the above potential risk factors, with the OR for urinary Atyp.C being 3.488 (95% CI 2.28–5.337). The change in the OR for urinary Atyp.C remained stable after multi-factor adjustment. The adjusted risk factors were verified after stratification of the pathological diagnosis, and only the OR for urinary Atyp.C was always greater than 1, which was statistically significant. Higher ORs for urinary Atyp.C levels correlated with a greater risk of increasing grade or invasion. In conclusion, the increase in urinary Atyp.C values was an independent risk factor for BC in urinary sediment analysis, and the levels of urinary Atyp.C positively correlated with the degree of grade or invasion.

This study had some limitations that should be acknowledged. First, only Atyp.C was analyzed as a risk factor for BC, with other known risk factors for BC, such as smoking, heavy use of antibiotics, and common history, omitted. Moreover, urinary Atyp.C values were easily affected by high squamous cell counts and pyuria, and its repeatability was unstable owing to different urinary sample qualities and clinical treatments and interventions in the same patient^15,16^. Therefore, using urinary Atyp.C levels alone as a diagnostic index is difficult. However, the construction of a diagnostic model combining urinary Atyp.C values with other risk factors may reduce the uncertainty from its influencing factors and improve diagnostic stability. Hence, we will focus on this in future research.

In summary, this study demonstrated that urinary Atyp.C was significantly elevated in patients with BC and was associated with tumor grade and invasion. Elevated Atyp.C levels may be an independent risk factor in patients with BC. Our results provide a potentially useful diagnostic indicator for the early diagnosis of patients with BC and suggest that urinary Atyp.C may be an important diagnostic tool that deserves further investigation. At the same time, urinary Atyp.C is one of the routine urine analyzer tests. If used as a diagnostic indicator, its detection method is convenient, and its affordability and stability will be better than those of urine cell morphology detection, which is more conducive to widespread promotion. Moreover, it will also improve early tumor detection in affected patients.

### Supplementary Information

Supplementary Information.

## Data Availability

The datasets generated during and/or analyzed during the current study are available from the corresponding author upon reasonable request.
